# Potential Antidiabetic Inhibitors and Antimicrobials from the Fungus *Fusarium lichenicola* Isolated from the Cicada *Cryptotympana mandarina* Distant, 1891

**DOI:** 10.4014/jmb.2507.07025

**Published:** 2025-12-09

**Authors:** Ton That Huu Dat, Vu Thi Thanh Tam, Le Canh Viet Cuong, Pham Hong Thai, Nguyen Thi Thanh Hai, Phan Tu Quy, Nguyen Thi Ai Nhung

**Affiliations:** 1 Mientrung Institute for Scientific Research, Vietnam National Museum of Nature, Viet Nam Academy of Science and Technology (VAST), 321 Huynh Thuc Khang, Hue City 49100, Vietnam; 2 Department of Chemistry, University of Sciences, Hue University, 77 Nguyen Hue, Hue city 49100, Vietnam; 3 Department of Natural Sciences & Technology, Tay Nguyen University, 567 Le Duan, Buon Ma Thuot, Dak Lak 63000, Vietnam

**Keywords:** *Fusarium lichenicola*, antimicrobials, antidiabetics, molecular docking, cicada, GC-MS

## Abstract

Fungi are among the main producers of diverse bioactive secondary metabolites. This study aimed to characterize the chemical constituents and evaluate the antimicrobial and antidiabetic activities of metabolites produced by the fungal *Fusarium lichenicola* isolated from the cicada *Cryptotympana mandarina*. Gas chromatography–mass spectrometry (GC-MS) analysis revealed that the fungal ethyl acetate extract contained 23 metabolites, including hydrocarbons, esters, terpenoids, alcohols and ketones, fatty acids, aldehydes, phenolics, and sulfur-containing compounds. *In vitro* assays indicated the fungal extract exhibited weak antimicrobial activity against Gram-positive and Gram-negative bacteria, as well as pathogenic fungi, with minimum inhibitory concentrations (MICs) ranging from 128 to 512 μg/ml. Antidiabetic potential of the fungal extract was demonstrated by inhibition of α-amylase and α-glucosidase enzymes, with IC_50_ values of 1202 ± 35 to 1347 ± 40 μg/ml, whereas representative metabolites demonstrated activities in the range of 285 ± 32 to 892 ± 85 μM. Molecular docking simulation identified the potential antidiabetic metabolites from the fungal extract. Further analyses revealed moderate correlation between docking scores and experimental activities, with regression R^2^ values of 0.58-0.62 and Pearson correlation coefficients of 0.76-0.79. These results highlight the predictive value of docking for identifying active metabolites, while emphasizing the necessity of experimental confirmation. Overall, these findings suggest that *F. lichenicola* derived from a cicada host represents a potential source of antidiabetic agents.

## Introduction

Diabetes is a chronic metabolic disorder characterized by hyperglycemia resulting from dysfunctional β-cell biology and impaired insulin action. Diabetes mellitus, particularly type 2 diabetes (T2D), has become a global health crisis, with over 800 million adults affected worldwide—a fourfold increase since 1990 [1]. According to the latest estimates, the number of people with diabetes is expected to double globally by 2050, from 529 million in 2021 to at least 1.3 billion. The number of people with type 2 diabetes will increase significantly, especially in areas with high obesity rates [2]. According to estimates from the Global Burden of Diseases, Injuries, and Risk Factors Study (GBD) 2019, diabetes was the eighth leading cause of combined death and disability worldwide [3], accounting for global health expenditures of approximately USD 966 billion, and forecast to exceed USD 1,054 billion by 2045 [4]. Characterized by chronic hyperglycemia, T2D is associated with severe complications such as retinopathy, nephropathy, and neuropathy [5]. These are microvascular complications that result from damage to small blood vessels due to sustained high blood sugar levels [6]. Current anti-diabetes drugs, such as metformin, rosiglitazone, sulfonylurea, acarbose and miglitol, are limited by side effects and suboptimal efficacy [7]. This underscores the urgent need for novel therapeutic agents with enhanced specificity and reduced adverse effects.

Fungi have evolved various symbiotic relationships with insects, ranging from mutualistic to parasitic [8]. Many fungi play a significant role in the lives of many insects, forming symbiotic relationships where both benefits. These fungi produce a variety of natural products, including enzymes, antimicrobial compounds, and vitamins, which are crucial for the host insect's nutrition, defense, and detoxification of plant compounds [9]. A recent review indicates that microbes associated with insects produce a diverse array of natural products with bioactive properties such as antimicrobial, cytotoxic, and antiparasitic activities [10]. Among them, many compounds have unique structures, such as gerumycins, pederin, dinactin, and formicamycins, suggesting that the microbes associated with insects are a promising source of bioactive natural products [10].

Fungi have garnered attention as prolific sources of bioactive metabolites with diverse pharmacological activities. Among these, the genus *Fusarium* has been identified as a rich reservoir of compounds exhibiting significant biological properties [11-13]. A comprehensive review on the secondary metabolites derived from endophytic *Fusarium* species reports over 300 compounds isolated from the genus *Fusarium* during 1999–2022 [12]. This fungal genus produced various secondary metabolites such as sterols, polyketides, alkaloids, terpenes, peptides and other compounds, with promising bioactivity such as antimicrobial, antiviral, anticancer, antioxidative, anti-parasitic and immunomodulatory activities [12]. Another review reports nearly 200 antimicrobial compounds produced by *Fusarium* [11]. Notably, recent updates reported 276 novel metabolites from at least 21 *Fusarium* species in the period of 2019 to 2024 [13]. Among them, many compounds showed potent antimicrobial and antidiabetic activities. For example, *F. equiseti* isolated from the leaf of *Gymnema sylvestre* produced a mycosterol with potent α-amylase and α-glucosidase inhibition activity with IC_50_ of 4.22 and 69.72μg/ml, compared with acarbose with IC_50_ of 5.75 and 55.29μg/ml, respectively [14]. Similarly, an anthraquinone, (S)-1,3,6-trihydroxy-7-(1-hydroxyethyl)-anthracene-9,10-dione, produced by *F. incarnatum* exhibited a strong inhibitory effect against α-glucosidase (IC_50_ = 77.67 ± 0.67 μΜ), compared with acarbose (IC_50_ = 711.8 ± 5 μΜ). Besides, docking simulations predicted this compound inhibited α-glucosidase substrate complex by binding Gln322, Gly306, Thr307, and Ser329 through hydrogen-bond interactions, suggesting that this anthraquinone can be considered a lead compound for further modifications and the development of a new effective drug candidate in the treatment of type 2 diabetes mellitus [15].

Cicadas, insects known for their unique life cycles and ecological roles, host a variety of microbial species. These fungi may produce novel metabolites with potential therapeutic properties. However, the antimicrobial and antidiabetic potential of fungi associated with cicadas remains largely unexplored. This study aims to investigate the antidiabetic and antimicrobial activity of *Fusarium lichenicola* isolated from the cicada *Cryptotympana mandarina*, contributing to the discovery of new natural products with potential therapeutic applications in diabetes and antimicrobial resistance management. To our knowledge, this is the first study to (i) characterize the chemical constituents of *F. lichenicola* and (ii) demonstrate its α-amylase and α-glucosidase inhibitory activity, especially from insect-derived fungi. Importantly, correlations between molecular docking predictions and experimental enzyme inhibition were investigated to evaluate the utility of *in silico* tools in natural product screening.

## Materials and Methods

### Isolation of the Fungus Strain

Cicada samples (*Cryptotympana mandarina*) were washed in running tap water three times to remove debris, dust, and other particles. Samples were then surface sterilized by immersion in 70% ethanol for 5 min, followed by 2% sodium hypochlorite for 10 min and 70% ethanol for 2 min before rinsing in distilled water three times. The sample (5 g) was crushed in 4.5 ml of sterile distilled water and subsequently homogenized through vortexing for one min. The suspension was diluted by a ten-fold dilution series to 10^-3^, and then aliquots of 50 μl were spread on Petri dishes containing Potato Dextrose Agar (PDA, Himedia, India). The plates were incubated at 25°C for 5-7 days. The colony of fungus VSV_15 was subcultured onto fresh PDA for purification and morphology analysis.

### Identification of the Fungus Strain

The genomic DNA of the fungus VSV_15 was extracted using the Wizard Genomic DNA Purification Kit (Promega, USA), and then the ITS gene sequence of the fungus VSV_15 was amplified using primers ITS4 and ITS5 [16]. The sequence was analysed by BioEdit v.2.7.5 and subsequently compared with ITS gene sequences available in the GenBank database through the NCBI Blastn program. The sequences were aligned using the ClustalW program in MEGA 12.0.11 and the phylogeny was constructed by the Neighbor-Joining (NJ) analysis using MEGA 12.0.11, with the robustness of the phylogeny evaluated through 1000 bootstrap replications. The ITS gene sequence of the fungus VSV_15 was deposited on GenBank under accession number PV888707.

### Fermentation and Ethyl Acetate Extraction

The fungus strains were cultured in 500 ml Potato Dextrose Broth (PDB, Himedia, India) for 7 days at 25°C under the shaking condition at 150 rpm, and the cultures were then centrifuged at 10,000 rpm for 10 min. The cell-free supernatants were extracted with ethyl acetate (1:1 v/v, 3 times) overnight at room temperature, and the ethyl acetate extractions were then evaporated under the reduced pressure for 12–24 h at 50°C to remove ethyl acetate and obtain the crude extracts.

### Gas Chromatography-Mass Spectrometry (GC-MS) Analysis

GC–MS analysis was performed using an Agilent 7890B gas chromatograph coupled with an Agilent 5977A mass detector (Agilent Technologies, USA) [17]. Separation was achieved with an HP-5MS capillary column (30 m × 0.250 mm × 0.25 μm film thickness; Agilent Technologies). The sample (1 μl) was injected in splitless mode with a flow rate of 1 ml/min. Helium (99.999% purity) was used as the carrier gas at 1 ml/min. The oven temperature was initially set at 60°C for 2 min, then increased to 260°C at 5°C/min and held for 1 min. The inlet and ionization source temperatures were set at 260°C and 280°C, respectively. The solvent delay was 3.00 min. The MS detector was operated in electron ionization (EI) mode at 70 eV, scanning the mass-to-charge (*m/z*) range of 50–550 in full scan mode. Data was processed using Agilent ChemStation software C.01.10 (Agilent Technologies). Compound identification was performed by comparing the acquired mass spectra with reference spectra from the W8N08 and NIST08 libraries with a minimum similarity index threshold of 90%. In addition, retention indices (RI) were calculated using a homologous series of n-alkane standards (C7–C40 saturated alkanes, Sigma-Aldrich) analyzed under identical experimental conditions. The experimentally determined RI values were then compared with those reported in the NIST retention index database for further confirmation of compound identity. The relative percentage of each compound was calculated based on GC peak areas.

### Antidiabetic Assay

**α-Amylase inhibitory assay.** The α-amylase enzyme inhibitory activity of the DCM extract and reference compounds (obtained from Sigma-Aldrich, USA and Aladdin, China) was determined according to the method described by Dat *et al*. [18]. In brief, the reaction mixture consisting of 50 μl sample was incubated with 50 μl starch azure solution and 50 μl α-amylase (1.0 U/ml) in Tris-HCl buffer (pH 6.9) in 96-well plates at 37°C for 10 min. The reaction was stopped by adding 50 μl of 50% acetic acid, and then the absorbance of the reactions was recorded at 650 nm using an ELx800 absorbance microplate reader (BioTek Instruments, USA). The inhibition activity was calculated as follows: inhibition (%) = 100 × [1 − (As − Abs)/(Ac − Acb)]. Whereas is the absorbance of the sample, Asb is the absorbance of the sample blank, Ac is the absorbance of the control, and Acb is the absorbance of the control blank. Acarbose was used as a positive control.

**α-Glucosidase inhibitory assay.** α-Glucosidase enzyme inhibitory activity of the DCM extract and reference compounds (Sigma-Aldrich) was determined according to the method described by Dat *et al*. [18] In brief, the reaction mixture consisting of 50 μl sample was incubated with 50 μl α-glucosidase (0.5 U/ml) in 0.1 M potassium phosphate buffer (pH 6.8) in 96-well plates at 37°C for 10 min. The reaction was started by adding 50 μl of 5 mM 4-nitrophenyl β-D-glucopyranoside (pNPG), followed by incubation at 37°C for 30 min. The reaction was stopped by adding 50 μl of 0.2 M Na_2_CO_3_, and then the absorbance of reactions was recorded at 405 nm using an ELx800 absorbance microplate reader (BioTek Instruments, USA). The inhibition activity was calculated as follows: inhibition (%) = 100 × [1 − (As − Abs)/(Ac − Acb)]. Where As is the absorbance of the sample, Asb is the absorbance of the sample blank, Ac is the absorbance of the control, and Acb is the absorbance of the control blank. Acarbose was used as a positive control.

### Antimicrobial Assay

Antimicrobial activity of the fungal extract was tested against seven reference microorganisms, *i.e.*, *Salmonella enterica* ATCC 13076, *Escherichia coli* ATCC 25922, *Pseudomonas aeruginosa* ATCC 27853, *Enterococcus faecalis* ATCC 29212, *Bacillus cereus* ATCC 14579, *Staphylococcus aureus* ATCC 25923, and *Candida albicans* ATCC 10231 using the broth microdilution method as described by Dat *et al*. [19]. Briefly, 100 μl of the bacterial inoculum (1×10^6^ CFU/ml) was added to wells containing 100 μl of the extracts at a range of different concentrations in 96-well plates. The plate was incubated at 37°C for 24 h, the absorbance at 630 nm was then measured using ELx800 absorbance microplate reader (BioTek Instruments). Minimum inhibitory concentrations (MICs) of the antibacterial extracts were determined as the lowest concentration, at which there was no growth of the bacteria. For the yeast, 100 μl of the inoculum (2 to 5 × 10^5^ CFU/ml for yeast) was added to wells containing 100 μl of the extracts at a range of different concentrations in 96-well plates. The plate was incubated at 28°C for 48 h. MICs of the anti-yeast extracts were determined as the lowest concentration, at which there was no growth of the yeast by the absorbance at 530 nm using an ELx800 absorbance microplate reader (BioTek Instruments).

### Molecular Docking Simulation

Ligand-protein static inhibitability can be evaluated using Molecular Operating Environment (MOE) 2022.10 [20] based on the molecular docking technique. In a typical procedure, the simulation follows steps as described by [18]

(i) Input preparation: Crystal structures of α-amylase (PDB-7TAA; DOI: 10.2210/pdb7TAA/pdb) and α-glucosidase (PDB-3W37; DOI: 10.2210/pdb3W37/pdb) were downloaded from RCSB Protein Data Bank; active-gird range: 4.5 Å from amino acids; force field: MMFF94x; Tether-Receptor strength: 5000; energy resolution: 0.0001 kcal.mol^-1^.Å^-1^. Ligand structures were compounds identified from the fungal extract by GC-MS in this work; geometrical optimization: Conj Grad algorithm; energy-change termination: 0.0001 kcal.mol^-1^; charge assignment: Gasteiger-Huckel method.

(ii) Docking simulation: Ligand-protein interaction was simulated; number of retaining poses = 10; maximum solutions per iteratio*n* = 1000; maximum solutions per fragmentatio*n* = 200.

(iii) Re-docking iteration: The inhibitory components (ligand and protein) were separated, then re-docked. The accuracy of the docking protocol is justified if the RMSD values (docked and re-docked conformations) are all under 2 Å.

(iv) Theoretical interpretation: The primary parameters for inhibitory effectiveness are docking score (DS) energy, root-mean-square deviation (RMSD) value, and numbers of hydrophilic bindings (hydrogen-like bonds). Besides, ligand-protein interactions and in-pose arrangements were mapped and rendered in 2D and 3D visualization, respectively.

**Fig. 1:**
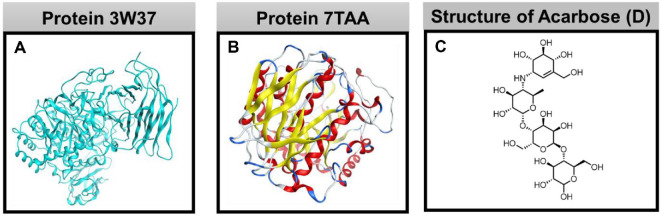
(A) Crystal structure of protein 3W37; (B) Crystal structure of protein 7TAA; (C) Structural formula of the control drug Acarbose (D).

**Fig. 2:**
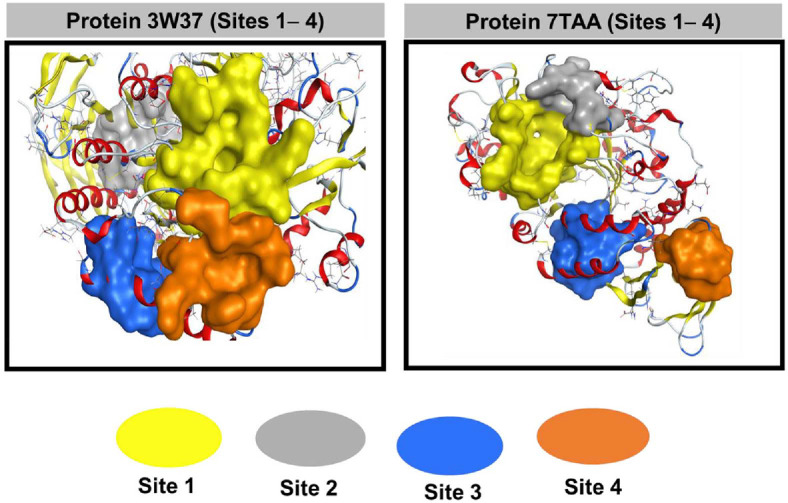
Quaternary structure of 3W37 and 7TAA with approachable sites by C1-C23 and D: site 1 (yellow), site 2 (grey), site 3 (blue), site 4 (orange).

Fig. 1 shows the crystal assemblies of the protein used in this work and the control drug (acarbose); the data was referenced from the RCSB Protein Data Bank. Fig. 2 presents the quaternary structure of 3W37 and 7TAA with approachable sites.

### QSARIS-Based Physicochemical Properties

The drug-likeness properties of phytochemicals of investigated compounds were screened and predicted by a combinational model, including (i) Parameters were the physical properties retrieved at https://admetlab3.scbdd.com along with polarizability calculated at, https://web.chemdoodle.com, the model was based on Gasteiger–Marsili method (Gasteiger & Marsili, 1980); (ii) Reference: Lipinski's rule of five [21]. The former includes molecular mass (Da), polarizability (Å^3^), size (Å), and dispersion coefficients (log*P* and log*S*); on the other side, the rule set criteria for a well membrane-permeable candidate are (i) molecular mass < 500 Da; (ii) hydrogen-bond donors ≤ 5; (iii) hydrogen-bond acceptors ≤ 10; (iv) log*P* < +5 [22, 23].

### ADMET-Based Pharmacokinetics and Pharmacology

ADMET properties (absorption, distribution, metabolism, excretion, and toxicity) were obtained from a web-based regressive model developed and maintained by the Molecular Modeling Group, Swiss Institute of Bioinformatics, *i.e.*, SwissADME (http://www.swissadme.ch/). The theoretical interpretations of output pharmacokinetic parameters were described by Pires *et al*. [24] and powered by the University of Melbourne and the University of Cambridge for public reference (http://biosig.unimelb.edu.au/pkcsm/theory).

### Statistical Analysis

All experiments were performed in triplicate, and results are expressed as mean values ± standard deviation (SD). The half-maximal inhibitory concentration (IC_50_) values were calculated using GraphPad Prism version 8.0 (GraphPad Software, USA). Correlation between docking score and experimental bioactivity (pIC_50_ = logIC_50_) was evaluated using regression and Pearson correlation coefficients.

## Results and Discussion

### Isolation and Identification the Fungus

**Fig. 3:**
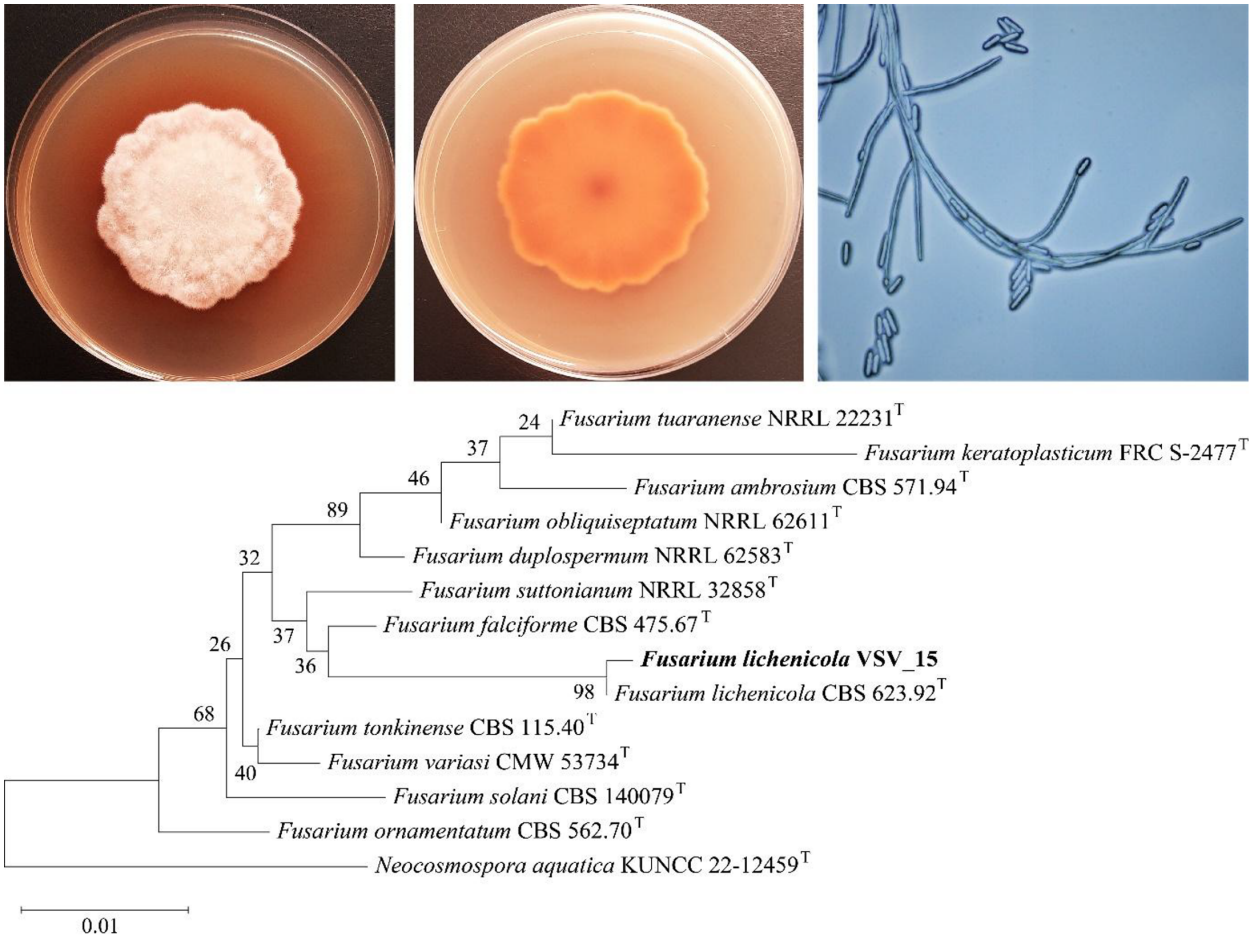
Macro- and micro-morphological characteristics, and phylogeny of the fungal strain VSV_15.

The fungus VSV_15 was isolated from the cicada *C. mandarina* collected in Hue City, Vietnam. The fungal colony grew well on PDA, reaching a diameter of 4.0–6.0 cm after 5-7 culture days at 25°C. The colony texture is aerial mycelium woolly/velvety, white growth with a pinkish-brown rim, whereas the reverse side is brown with a diffusible brown-red pigment (Fig. 3). Microscopic examination revealed slender, septate fungal hyphae. The macroconidia were ellipsoid to cylindrical with straight, smooth edges. Macroconidia were arranged both singly and in clusters. Numerous chlamydospores originating from short lateral branches on the hyphae were also observed (Fig. 3). These macroscopic and microscopic characteristics suggest that this fungus may be *Fusarium lichenicola*.

Identification of the fungus VSV_15 was further confirmed by ITS gene sequence analysis. The ITS rRNA gene sequence of VSV_15 had 100% identity with the ITS rRNA gene sequence of strain *F. lichenicola* CBS 623.92 (type strain). The ITS gene phylogeny revealed that the fungus VSV_15 was grouped in the clade of *F. lichenicola* (Fig. 3). The minor difference in branch length between these two strains on the phylogenetic tree is due to a difference in sequence length: 488 nt for strain VSV_15 and 448 nt for strain CBS 623.92. Therefore, the fungus VSV_15 was identified as *F. lichenicola*.

### Chemical Constituents from Ethyl Acetate of the Fungus

The GC-MS chromatogram of the fungal extract (Fig. S1) revealed multiple peaks, indicating the presence of diverse chemical constituents. Subsequent analysis based on library matches and retention index comparison with literature data identified a total of 23 compounds, which accounted for 79.8% of the total composition of the extract (Table 1). These identified compounds belong to several chemical classes, including hydrocarbons, esters, terpenoids, alcohols and ketones, fatty acids, aldehydes, phenolic and sulfur-containing compounds. Among these, 3-methylbutyl hexanoate (12.6%), ethyl octanoate (8.07%), *p*-xylene (7.77%), 3-mercaptohexyl acetate (5.79%), and bicycloelemene (5.06%) were the most abundant.

Although reviews have shown that *Fusarium* species produce a wide range of biologically active secondary metabolites [11-13], studies specifically focused on the secondary metabolites of *F. lichenicola* remain extremely limited. A recent investigation into the chemical composition and antibacterial activity of an ethyl acetate extract from *F. lichenicola* isolated from *Trigonella foenum-graecum* leaves identified 20 diverse secondary metabolites belonging to various chemical classes, including hydrocarbons, aromatic compounds, sulfur-containing heterocycles, esters, and nitrogenous derivatives [25]. Among them, the major compounds were identified, such as 2-((4-methylpentan-2-yloxy)carbonyl)benzoic acid (15.33%), pyrrolo[1,2-a]pyrazine-1,4-dione, hexahydro-3-(2-methylpropyl) (12.42%), and benzeneethanamine, 4-benzyloxy-2-fluoro-β-hydroxy-5-methoxy (3.45%) [25].

### Anti-Diabetic and Antimicrobial Activities of the Fungus

Antidiabetic assays demonstrated that the fungal extract exhibited inhibitory activity against α-amylase and α-glucosidase with IC_50_ of 1202 ± 35 and 1347 ± 40 μg/ml, respectively (Table 2). Additionally, antimicrobial testing revealed that the extract exhibited weak, broad-spectrum antimicrobial activity, with MIC values ranging from 128 to 512 μg/ml (Table 3). Notably, the extract exhibited the strongest activity against *S. aureus* and *E. coli* with MIC = 128 μg/ml, followed by *S. enterica*, *E. faecalis*, *B. cereus*, and *C. albicans* with MIC = 256 μg/ml, while a weaker effect was observed against *P. aeruginosa* with MIC = 512 μg/ml. These results suggest the presence of potential antidiabetic compounds in the fungal extract. Although antimicrobial activity of the extract is generally considered negligible in natural product screening, it does not preclude the presence of potent individual metabolites at low concentrations, which may be uncovered through bioassay-guided isolation. Currently, there are no reports on the antidiabetic potential of *F. lichenicola*, whereas its antimicrobial activity has been documented only in a recent study. The ethyl acetate extracts of this fungus showed antibacterial effects against *B. megaterium*, *S. aureus*, *E. coli*, and *S. typhi* with inhibition zones of 10-15 mm at a concentration of 100 μg/disc [25].

It is noted that many compounds identified from the fungal extract (Table 1) have been reported to show antimicrobial activity. For instance, formic acid exhibits effective antimicrobial activity against a broad spectrum of microorganisms, including *Salmonella* spp., *S. Typhimurium*, *Listeria monocytogenes*, *Campylobacter jejuni*, *Clostridium perfringens*, *E. coli*, and *S. aureus*, with MICs of 0.1-0.15 μM [26, 27]. Antimicrobial effects of formic acid primarily occur by disrupting cellular processes through penetration of the cell membrane, acidification of the cytoplasm, and interference with cellular metabolism [26, 28]. Another compound, 4-isopropenyl-1-methylcyclohexene (limonene), has already been proven to have outstanding antimicrobial activities against the Gram-positive bacteria *S. aureus* and *B. subtilis*, the Gram-negative bacterium *E. coli*, and the yeast *S. cerevisiae* with MICs of 0.5-1.0 μg/ml [29]. Dimethyltrisulfane, an organosulfur compound, has been reported to show promising antimicrobial activity against various bacterial pathogens, including *Enterobacter aerogenes*, *E. coli*, *S. enterica*, *Shigella sonnei*, *Listeria monocytogenes*, *Yersinia enterocolitica*, *S. aureus*, *Leuconostoc mesenteroides*, *Pediococcus pentosaceus*, and *Lactobacillus plantarum* [30]. Antimicrobial effects of dimethyltrisulfane are involved in membrane disruption, inhibition of germination, and potential interference with cellular processes [31-33]. n-Hexadecanoic acid (palmitic acid) has revealed good antibacterial effects on *S. aureus*, *B. subtilis*, *E. coli*, and *Klebsiella pneumoniae* [34]. Also, palmitoleic acid has displayed antimicrobial activity against various microbial pathogens, including Gram-positive and Gram-negative bacteria and fungi [35, 36]. These fatty acids may exhibit antimicrobial activity by disrupting bacterial cell membranes, inhibiting enzyme activity, inducing toxic peroxidation, and causing cell lysis [37-40]. Otherwise, the antidiabetic potential of the compounds identified in the fungal extract (Table 1) has been rarely documented in the existing literature. Notably, palmitoleic acid has demonstrated potential in enhancing insulin sensitivity and lowering the risk of diabetes [41-43]. Additionally, an *in silico* investigation suggests that palmitic acid may contribute to antidiabetic effects by inhibiting enzymes involved in insulin signaling such as protein tyrosine phosphatases [44]. Therefore, further bioassay-guided isolation, along with complementary *in silico* and *in vitro* investigations, is necessary to identify novel and promising antimicrobial and antidiabetic compounds produced by the fungus.

### Molecular Docking Simulation

GC-MS analysis and *in vitro* antidiabetic assay showed that the fungal extract exhibited potential antidiabetic activity and contained diverse compounds. Therefore, molecular docking simulation was performed to identify potential antidiabetic compounds in the fungal extract. The inhibitory ability of the compounds was evaluated through DS docking energy (kcal.mol^-1^) and the number of interactions formed between the compounds and the susceptible sites. Specifically, the lower the docking energy and the larger the number of interactions, the higher the inhibitory ability of the compound at that site.

The quaternary structure of 3W37 and 7TAA with approachable sites by **C1-C23** and control (**D**): site 1 (yellow), site 2 (grey), site 3 (blue), site 4 (orange) are presented in Fig. 2 and Table S1. The fact is that these accessible sites (1-4) play an important role in determining the interaction and inhibition capabilities of the compounds with respect to the two proteins (3W37 and 7TAA). The inhibition ability of the compounds was evaluated through two main criteria: docking score energy (DS kcal.mol^-1^) and the number of interactions formed between the compounds and the accessible sites. Specifically, the lower the docking energy and the larger the number of interactions, the higher the inhibitory ability of the compound at that position.

For the protein 3W37, after screening at docking sites, it was found that sites 1 (yellow) and 2 (grey) are favorable for docking (Table S1). These are sites with low docking energy and a large number of interactions, thereby supporting the docking process of proteins. Specifically, compounds **C1 - C9**, **C11 - C20**, **C23** and the control D favorably inhibit protein 3W37 at site 1 (yellow) and **C10**, **C21**, **C22** are favorable for docking at site 2 (grey). Docking energy values range from -7.0 kcal.mol^-1^ to -13.9 kcal.mol^-1^. For the 7TAA protein position 1 (yellow) has the lowest docking energy (-7.7 kcal.mol^-1^ to -12.5 kcal.mol^-1^) and has a number of interactions from 1 to 8 interactions (Table S1).

On the other hand, when examining the sizes of potential positions of proteins 3W37 and 7TAA, we found that among the 4 sites, sites 1 and 2 are much larger in size than sites 3 and 4 (Table S2). Specifically for the 3W37 protein, site 1 and site 2 have sizes of 63 Å and 37 Å, respectively. For the 7TAA protein, the size of site 1 is 107 Å. In addition, the amino acid residues at these positions are relatively large, so it is favorable for the interaction between compounds **C1-C23** with amino acids of proteins 3W37 and 7TAA. After determining the optimal site to approach proteins 3W37 and 7TAA, molecular docking simulations were conducted to determine in detail the docking energy and the types of bonds and interactions formed between the compounds and the inhibitory protein.

The results of docking simulation as docking energy (DS), RMSD index, van der Waals interactions, hydrogen bonds are presented in Table S3 and Figs. S2 and S3. All simulations had root mean square deviation (RMSD) values less than 2Å, suggesting that docking results are reliable [45]. By comparing the docking results such as DS, number of hydrogen bonds and van der Waals interactions, the ability to inhibit protein 3W37 of compounds were evaluated. The docking energy values ranged from -7.0 kcal.mol^-1^ to -13.9 kcal.mol^-1^ with the number of interactions from 0 to 5 interactions. In which, compound **C11** had a docking value of -13.9 kcal.mol^-1^ with the number of interactions of 7 (3 H-donors and 2 H-acceptors), which is better than the control drug Acarbose. In addition, by comparing the docking energy values, the number of hydrogen bonds and van der Waals interactions, potential inhibition of the compounds againts 3W37 by molecular docking simulation might accords with the order: **C11 > D > C23 > C12 C13 > C19 > C8 > C22 > C21 > C15 > C6 » C16 > C5 > C9 > C10 > C17 > C4 » C18 > C1 > C3 » C20 > C14 > C7 > C2** (Table S3).

For the protein 7TAA, the docking results such as docking energy (DS), RMSD index, van der Waals interactions, hydrogen bonds are presented in Table S4 and Figs. S4 and S5. The simulation results had root meaning square deviation (RMSD) values less than 2Å, suggesting that the docking results are reliable [45]. The docking results revelaed that compound **C9** has the best ability to inhibit 7TAA protein. The potential stability of the remaining complexes by molecular docking simulation might accords with the order: **D > C9 > C3 > C14 » C21 > C16 > C5 > C13 » C22 > C23 > C7 > C6 > C11 > C8 » C19 > C15 > C17 > C2 > C1 > C4 > C10 > C12 » C18 > C20** (Table S4).

Overall, the compounds that showed strong inhibition of the two target proteins, 3W37 and 7TAA, often had a ring structure and contained active functional groups such as hydroxy (-OH), carbonyl (-C=O), ester (-COO-), ketone (-C(=O)-), carboxylic acid (-COOH), thiol (-SH) and disulfide (-S-S-). These functional groups play an important role in forming interactions with amino acids located in the active site of the protein. From the above observations, an initial assessment can be made that the components in the extract have the potential to become potential inhibitors for two representative proteins, 4W93 and 3W37, in the molecular docking simulation study.

### QSARIS-Based Physicochemical Properties

Physicochemical parameters of the compounds **C1-C23** and the reference drug Acarbose (**D**) are present in Table S5, including the mean inhibition energy (DSTB, kcal.mol^-1^); molecular weight (Ma, amu); polarity (Å3); volume (size) (Å), and dispersion coefficients LogP, LogS. The parameters were calculated using the Gasteiger-Marsili method [46]. The biological or pharmacological activity of the studied compounds was evaluated based on Lipinski's rule of five, a reliable criterion for predicting the potential of a compound as a drug [21]. The rule states that an analyte with good inhibitory ability through interaction with amino acids should have LogP < 5, molecular weight ≤ 500 amu with the number of hydrogen bond acceptors ≤ 10, the number of hydrogen bond donors ≤ 5 [22]. The results present in Table S5 indicated that all compounds **C1-C23** had molecular weights below 500 amu, logP < 5 for the dispersion coefficient, and the solubility coefficient has negative values. In addition, **C1-C23** have large polarity values > 10 Å^3^ and lead to high binding ability with amino acids through showing strong hydrogen bonding and van der interactions, thereby being very beneficial for protein inhibition. These findings suggested that compounds **C1-C23** have the potential to be compatible with pharmaceutical applications in physiological environments in the treatment of new diseases.

### Correlation between Molecular Docking Predictions and Experimental Bioactivity

To validate the molecular docking predictions, selected reference compounds were subjected to *in vitro* evaluation of their antidiabetic activities (Table 4). The bioassays demonstrated inhibitory activities against both α-amylase and α-glucosidase, with IC_50_ values ranging from 288 ± 26 to 892 ± 85 and 285 ± 32 to 765 ± 85 μM, respectively, compared to acarbose (IC_50_ values of 166 ± 4 and 183 ± 7 μM, respectively). The minor difference in the IC_50_ values of acarbose reported in Tables 2 and 4 is due to variations between independent experiments. A general trend was observed between docking scores and experimental activity, wherein compounds with more favorable docking scores (*i.e.*, more negative) tended to exhibit lower IC_50_ values. This relationship underscores the utility of molecular docking as a predictive tool for identifying potential inhibitors. Nonetheless, the correlation was not strictly linear, likely reflecting additional factors not captured by docking simulations, such as aqueous solubility, membrane permeability, and conformational adaptability of the target proteins under physiological conditions.

Further correlation analyses showed moderate regression between docking scores and experimental activities, with R^2^ values of 0.62 and 0.58 for α-amylase and α-glucosidase inhibitions, respectively and corresponding Pearson correlation coefficients of 0.79 (*p* = 0.06) and 0.76 (*p* = 0.03), respectively (Fig. S6). Comparable studies have reported similar levels of regression, with R^2^ ranging from 0.552 to 0.981, although Pearson correlation coefficients varied more widely (r = 0.35–0.74) [47-51]. Such variability among studies may be attributed to the intrinsic limitations of docking scores, which are derived from simplified, approximate models that frequently neglect critical factors such as protein flexibility, solvation effects, and entropic contributions [52]. In contrast, experimental bioactivities reflect the complex biochemical behavior of ligand–protein interactions under dynamic assay conditions. These findings indicate that while docking provides valuable predictive insights for potential inhibitors, the observed discrepancies emphasize the necessity of employing integrated approaches that combine computational predictions with experimental validation to reliably identify and optimize lead compounds.

### ADMET-Based Pharmacokinetics and Pharmacology

To analyze the pharmacokinetic-toxicological properties of compounds **C1-C23** and the reference drug Acarbose using the SwissADME regression model involving five parameters: absorption, distribution, metabolism, excretion and toxicity, all of which play an important role in predicting the potential application of natural products in drug development. The ADMET analyses are presented in Tables S6 and S7. Regarding absorption, two important parameters that determine the absorption of a compound are the absorption in the human intestine and the permeability of the colon carcinoma cell line (Caco2). A substance is considered poorly absorbed if the percentage absorbed in the human intestine is less than 30%. In general, compounds **C1-C23** have high intestinal absorption rates and high Caco2 permeability (log Papp > 0.9). The distribution of compounds **C1-C23** depends on many parameters, and the results indicate that these compounds tend to accumulate in body parts, easily pass through the blood-brain barrier and have low penetration into the central nervous system. In terms of metabolism, compounds **C1-C23** and the control drug Acarbose have the ability to inhibit different types of substrates (Table S7 and S8). In terms of toxicity, the components in the extract are safe for the body, specifically:(i) not carcinogenic (AMES); (ii) do not block potassium channels (inhibit hERG I and II); (iii) do not cause liver toxicity. In summary, the ADMET parameters of compounds **C1-C23** are all within the allowable range of solubility, membrane permeability, skin permeability, substrate specificity, metabolism, clearance and toxicity (low). This confirms the potential compounds in the preparation of "drug-like" compounds to guide further experimental studies. However, it should be noted that the molecular docking and ADMET analyses reported here are predictive in nature. While such *in silico* approaches provide useful preliminary insights into potential bioactivity, the results are not definitive and require further confirmation through *in vitro* assays and, ultimately, *in vivo* studies.

## Conclusion

In the present study, chemical constituents and antimicrobial and antidiabetic activities of the ethyl acetate extract derived from the fungus *F. lichenicola* isolated from the cicada *C. mandarina* were investigated. GC-MS analysis identified 23 metabolites from the fungal ethyl acetate extract, including hydrocarbons, esters, terpenoids, alcohols and ketones, fatty acids, aldehydes, phenolics, and sulfur-containing compounds. *In vitro* assays indicated the fungal extract exhibited weak antimicrobial activity against a broad spectrum of pathogenic microorganisms with MICs of 128 - 512 μg/ml. Antidiabetic potential of the fungal extract was demonstrated by inhibition of α-amylase and α-glucosidase enzymes, with IC_50_ values of 1202 ± 35 to 1347 ± 40 μg/ml, whereas representative metabolites demonstrated activities in the range of 285 ± 32 to 892 ± 85 μM. Molecular docking simulation identified the potential antidiabetic metabolites from the fungal extract. Further analyses revealed moderate correlation between docking scores and experimental activities, highlighting the predictive value of docking for identifying active metabolites, while underscoring the need for experimental validation. These findings suggest that *F. lichenicola* derived from a cicada host represents a natural source of compounds with potential antidiabetic activity. Future work should focus on bioassay-guided isolation of active compounds, cytotoxicity evaluation, and *in vivo* validation to confirm the therapeutic potential of *F. lichenicola* metabolites.

## Supplemental Materials

Supplementary data for this paper are available on-line only at http://jmb.or.kr.



## Figures and Tables

**Table 1:** Chemical constituents in the fungal extract.

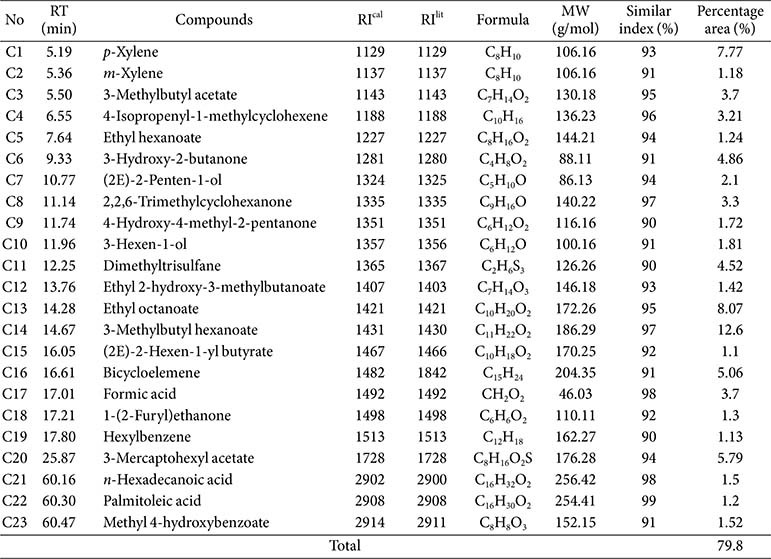

**Table 2:** Antidiabetic activity of the fungal extract.

Sample	IC_50_, μg/ml	
	α-Amylase inhibition	α-Glucosidase inhibition
Fungal extract	1202 ± 35 1347 ± 40
Acarbose	105 ± 3 121 ± 4

**Table 3:** Antimicrobial activity of the fungal extract.

Microorganisms	MIC (μg/ml)
*Escherichia coli*	128
*Salmonella enterica*	256
*Pseudomonas aeruginosa*	512
*Enterococcus faecalis*	256
*Staphylococcus aureus*	128
*Bacillus cereus*	256
*Candida albicans*	256

**Table 4:** Antidiabetic activity of the reference compounds.

No	Compounds	IC_50_, μM	
		α-Amylase inhibition	α-Glucosidase inhibition
C1	*p*-Xylene	>1,000	>1,000
C4	4-Isopropenyl-1-methylcyclohexene	561 ± 49	765 ± 85
C5	Ethyl hexanoate	892 ± 85	587 ± 65
C11	Dimethyl trisulfide	332 ± 23	462 ± 44
C13	Ethyl octanoate	502 ± 54	574 ± 66
C21	*n*-Hexadecanoic acid	>1,000	285 ± 32
C22	Palmitoleic acid	>1,000	303 ± 20
C23	Methyl 4-hydroxybenzoate	288 ± 26	511 ± 32
D	Acarbose	166 ± 4	183 ± 7
