# Supplementary material for: Potential Antidiabetic Inhibitors and Antimicrobials from the Fungus Fusarium lichenicola Isolated from the Cicada Cryptotympana mandarina Distant, 1891
Source: J Microbiol Biotechnol. 2025 Dec 9;35:e2507025. doi: 10.4014/jmb.2507.07025 (PMC12706144; doi:10.4014/jmb.2507.07025)
Supplement: Supplementary file 1 [file jmb-35-e2507025-supple.pdf]

J. Microbiol. Biotechnol. 2025. 35: e2507025

https://doi.org/10.4014/jmb.2507.07025

Supplementary Figures and Tables

Potential antidiabetic inhibitors and antimicrobials from the fungus *Fusarium lichenicola* isolated from the cicada *Cryptotympana mandarina* Distant, 1891

Ton That Huu Dat[1*], Vu Thi Thanh Tam[1], Le Canh Viet Cuong[1], Pham Hong Thai[1], Nguyen Thi Thanh Hai[2], Phan Tu Quy[3], Nguyen Thi Ai Nhung[2*]

[1]Mientrung Institute for Scientific Research, Vietnam National Museum of Nature, Viet Nam Academy of Science and Technology (VAST), 321 Huynh Thuc Khang, Hue City 49100, Vietnam

[2]Department of Chemistry, University of Sciences, Hue University, 77 Nguyen Hue, Hue city 49100, Viet Nam

[3]Department of Natural Sciences & Technology, Tay Nguyen University, 567 Le Duan, Buon Ma Thuot, Dak Lak 63000, Viet Nam

Table S1. Prescreening results on inhibitability of ligands (**C1-C23**) and controlled drug **(D)** towards the sites of protein **3W37** and protein **7TAA.**

**P**	**C**	**Site 1**		**Site 2**		**Site 3**		**Site 4**	
		E	N	E	N	E	N	E	N
3W37	**C1**	**-8.1**	**1**	-7.5	0	-7.0	0	-6.8	0
	**C2**	**-7.0**	**0**	-6.7	0	-6.0	0	-6.6	0
	**C3**	**-8.0**	**1**	-7.3	0	-7.8	1	-6.9	0
	**C4**	**-8.2**	**1**	-7.1	0	-7.0	0	-6.4	0
	**C5**	**-9.0**	**2**	-8.3	1	-7.8	1	-7.4	0
	**C6**	**-9.1**	**2**	-8.0	1	-7.5	0	-7.0	1
	**C7**	**-7.8**	**1**	-6.7	0	-6.9	0	-6.1	0
	**C8**	**-11.2**	**3**	-8.9	1	-8.5	1	-8.2	1
	**C9**	**-8.9**	**2**	-7.1	0	-8.0	1	-7.4	1
	**C10**	-8.0	1	**-8.7**	**2**	-7.3	0	-7.7	1
	**C11**	**-13.9**	**7**	-10.9	4	-10.4	3	-10.5	3
	**C12**	**-12.3**	**4**	-10.1	2	-9.7	2	-8.3	1
	**C13**	**-12.1**	**4**	-7.8	1	-10.0	2	-7.2	1
	**C14**	**-7.9**	**1**	-6.7	0	-6.5	0	-6.2	0
	**C15**	**-9.2**	**2**	-7.3	0	-7.0	0	-6.8	0
	**C16**	**-9.1**	**2**	-8.1	1	-7.5	0	-7.2	0
	**C17**	**-8.3**	**1**	-7.4	0	-7.0	0	-6.6	0
	**C18**	**-8.2**	**1**	-6.8	0	-6.5	0	-6.0	0
	**C19**	**-11.8**	**3**	-7.2	1	-6.9	1	-6.3	0
	**C20**	**-8.0**	**1**	-6.7	0	-6.4	0	-6.0	0
	**C21**	-8.3	1	**-9.3**	2	-7.6	0	-7.2	0
	**C22**	-8.0	1	**-9.5**	2	-8.4	1	-7.6	0
	**C23**	**-13.0**	**4**	-10.9	2	-11.0	2	-9.7	1
	**D**	**-13.4**	**5**	-11.5	3	-10.6	2	-9.6	2
7TAA	**C1**	**-8.0**	**1**	-6.4	0	-6.0	0	-6.3	0
	**C2**	**-8.2**	**1**	-7.0	0	-6.7	0	-6.2	0
	**C3**	**-12.0**	**4**	-10.1	2	-8.5	1	-7.8	1
	**C4**	**-7.9**	**1**	-6.7	0	-6.3	0	-6.0	0
	**C5**	**-11.0**	**3**	-8.9	1	-9.0	1	-8.6	1
	**C6**	**-9.2**	**2**	-8.4	1	-7.7	0	-8.1	1
	**C7**	**-9.3**	**2**	-8.0	1	-7.4	1	-7.2	0
	**C8**	**-9.0**	**2**	-7.9	1	-7.3	1	-6.7	0
	**C9**	**-12.1**	**4**	-10.3	2	-10.6	2	-8.9	1
	**C10**	**-7.9**	**1**	-6.9	0	-6.7	0	-6.2	0
	**C11**	**-9.1**	**2**	-8.1	1	-6.6	0	-6.9	0
	**C12**	**-7.8**	**1**	-6.8	0	-6.5	0	-6.7	0
	**C13**	**-11.0**	**3**	-8.1	1	-7.9	1	-7.2	0
	**C14**	**-11.3**	**3**	-8.3	1	-7.0	0	-6.7	0
	**C15**	**-8.9**	**2**	-7.4	1	-6.9	0	-6.4	0
	**C16**	**-11.2**	**3**	-6.8	1	-7.0	1	-6.1	0
	**C17**	**-8.8**	**2**	-6.2	0	-7.1	1	-6.0	0
	**C18**	**-7.8**	**1**	-7.0	0	-6.9	0	-6.3	0
	**C19**	**-9.0**	**2**	-7.7	1	-7.5	1	-6.4	0
	**C20**	**-7.7**	**1**	-6.8	0	-6.4	0	-6.1	0
	**C21**	**-11.3**	**3**	-7.1	1	-7.0	1	-6.6	0
	**C22**	**-11.0**	**3**	-6.5	0	-7.1	1	-6.3	0
	**C23**	**-9.5**	**2**	-7.0	0	-6.9	0	6.3	0
	**D**	**-12.5**	**4**	-9.4	2	-8.7	1	-8.3	1

Table S2. Size with amino acid residues at the surveyed positions of proteins **3W37** and **7TAA.**

**Protein**	**Site**	**Size (Å)**	**Residues**
**3W37**	1	63	Asp232 Ile233 Ala234 Phe236 Tyr243 Trp329 Asp357 Ile358 Ile396 Trp432 Trp467 Asp469 Met470 Phe476 Arg552 Trp565 Gly567 Asp568 Phe601 Arg624 His626
	2	37	Glu301 Tyr659 Thr662 Leu663 Asp666 Arg670 Arg676 Gly698 Arg699 Gly700 Asn758 Ile759 Val760 Ala761 Gly791 Glu792
	3	19	Val341 Asp342 Tyr344 Ala345 Ile349 Pro350 Leu351 Lys385 Asn389 Gln391 Met767 Thr768
	4	23	Tyr331 Arg332 Ile358 Asp359 Met361 Ala363 Phe364 Asp370 His373 Phe374 Arg629
**7TAA**	1	107	His80 Tyr82 Trp83 His122 Tyr155 Leu166 Gly167 Leu173 Arg204 Asp206 Thr207 Val208 Lys209 His210 Glu230 Val231 Leu232 His296 Asp297 Asp340 Arg344
	2	29	Gln35 Tyr75 Gly76 Asp77 Tyr79 His80 Trp83 Asp340 Pro341 Arg344
	3	35	Asn251 Ile254 Ile276 Val279 Lys280 Ser286 Leu289 Gly290 Thr291 Phe317 Asn321 Asp322 Gly323 Lys383 Asn384
	4	27	Ser410 Lys412 Gly416 Asp417 Tyr419 Met455 Ala456 Gly457 Gly458 Leu459

Table S3. Detailed molecular docking simulation results for ligands (**C1-C23** and **D**)-**3W37** inhibitory complexes.

Name	DS	RMSD	Hydrogen bond L	P		T	D	E	van der Waals interaction
**C1-3W37**	-8.1	1.08	C	6-ring	Trp 329	H-pi	3.85	-0.6	Trp 432, Asp 568, Ile 396, Trp 467, Arg 552, Asp 469, Asp 357, Phe 601, Met 470
**C2-3W37**	-7.0	1.57	-	-	-	-	-	-	Arg 552, Trp 565, Asp 568, Asp 469, Met 470, Trp 329, Trp 432, His 626, Phe 601, Ile 358, Trp 467, Asp 357
**C3-3W37**	-8.0	0.62	C	6-ring	Trp 329	H-pi	3.80	-0.6	Asp 469, Met 470, Trp 432, Asp 357, Asp 568, Phe 601, His 626, Gly 567, Ile 396, Trp 467, Trp 565, Arg 552
**C4-3W37**	-8.2	0.41	C	6-ring	Trp 329	H-pi	4.00	-0.7	Arg 552, Gly 567, Asp 568, Trp 565, Asp 357, Trp 467, Phe 601, Ile 396, Ile 358, Trp 432, Asp 469, His 626, Met 470
**C5-3W37**	-9.0	1.28	O	S	Met 470	H-donor	3.32	-0.1	Asp 469, Asp 357, Asp 568, Trp 431, Asp 232, Trp 565, Trp 467, His 626, Arg 552
			C	6-ring	Phe 601	H-pi	3.60	-0.7	
**C6-3W37**	-9.1	1.3	O	O	Asp 357	H-donor	2.96	-3.6	Gly 567, Trp 467, Arg 552, Trp 565, Asp 469, Met 470, Phe 601, Arg 624, Ile 396, Asp 568
			O	N	His 626	H-acceptor	2.99	-0.9	
**C7-3W37**	-7.8	0.35	O	O	Asp 357	H-donor	2.87	-2.9	Ile 358, Trp 329, Met 470, Ile 396, Asp 568, Asp 469, Gly 567, Arg 552, Trp 565, Trp 432, Phe 601
**C8-3W37**	-11.2	1.12	O	N	His 626	H-acceptor	3.61	-0.6	Met 470, Arg 552, Gly 567, Trp 432, Asp 568, Trp 565, Asp 357, Ile 396, Trp 467, Asp 469
			C	6-ring	Trp 329	H-pi	4.29	0.6	
			C	6-ring	Phe 601	H-pi	3.52	-0.6	
**C9-3W37**	-8.9	0.89	O	N	Trp 432	H-acceptor	3.14	-0.7	Trp 329, Asp 568, Phe 601, Gly 67, Trp 565, Asp 357, Asp 469, Ile 396, Met 470, Trp 467, Arg 552
			O	N	His 626	H-acceptor	3.02	-2.5	
**C10-3W37**	-8.7	0.62	O	O	Ile 759	H-donor	2.99	-1.1	Tyr 659, Arg 699, Leu 663, Thr 662, Gly 791, Arg 670, Asp 666, Thr 790, Asn 758
			O	N	Glu 792	H-acceptor	3.13	-1.7	
**C11-3W37**	-13.9	1.78	S	O	Asp 357	H-donor	3.89	-0.4	Trp 329, Phe 601, Trp 565, Gly 567
			S	S	Met 470	H-donor	3.76	0.1	
			S	O	Asp 469	H-donor	3.43	-0.9	
			S	O	Asp 568	H-donor	3.70	-1.0	
			S	O	Asp 568	H-donor	4.24	-0.5	
			S	N	Trp 432	H-acceptor	3.36	-1.6	
			S	C	Arg 552	H-acceptor	3.78	-1.0	
**C12-3W37**	-12.3	0.97	O	O	Áp 568	H-donor	3.26	-1.3	Asp 469, His 626, Ile 396, Trp 467, Trp 565, Arg 624, Gly 567, Arg 552, Asp 357, Trp 329, Ile 358
			O	S	Met 470	H-donor	3.43	-0.3	
			C	S	Met 470	H-donor	3.90	-0.7	
			C	6-ring	Phe 601	H-pi	3.61	-0.7	
**C13-3W37**	-12.1	0.63	O	S	Met 470	H-donor	3.84	-0.5	Trp 432, Ile 396, Arg 552, Gly 567, Trp 467, His 626, Phe 601, Arg 624, Asp 357, Trp 565
			C	O	Asp 568	H-donor	3.32	-0.7	
			C	5-ring	Trp 329	H-pi	4.37	-0.6	
			C	6-ring	Trp 329	H-pi	3.78	-1.3	
**C14-3W37**	-7.9	1.38	C	S	Met 470	H-donor	3.95	-0.7	His 626, Trp 565, Gly 567, Asp 568, Asp 232, Phe 601, Arg 552, Trp 329, Trp 432, Asp 357, Asp 469, Ile 396, Trp 467
**C15-3W37**	-9.2	1.16	O	N	Trp 432	H-acceptor	3.20	-0.7	Gly 567, Trp 565, Asp 568, Asp 232, Phe 601, Arg 552, Ile 396, Asp 357, Trp 467, Met 470, Asp 469
			C	6-ring	Trp 329	H-pi	3.82	-0.9	
**C16-3W27**	-9.1	1.64	O	O	Asp 357	H-donor	2.94	-7.2	Asp 469, Trp 565, Arg 624, Trp 467, Ile 396, Phe 601
			O	N	His 626	H-acceptor	3.19	-1.1	
**C17-3W37**	-8.3	0.82	O	N	His 626	H-acceptor	3.11	-0.7	Trp 467, Ile 396, Asp 469, Met 470, Asp 568, Trp 432, Phe 601, Gly 567, Arg 552, Trp 565, Arg 624, Asp 357
**C18-3W37**	-8.2	1.58	C	6-ring	Trp 329	H-pi	3.85	-0.6	Asp 469, Asp 568, Met 470, Phe 236, Trp 432, Asp 630, Ala 602, Ala 628, Phe 601, Arg 552, Asp 357, Ile 396
**C19-3W37**	-11.8	1.08	S	O	Asp 357	H-donor	3.60	-1.1	
			S	S	Met 470	H-donor	3.79	-0.9	

			S	N	Trp 432	H-acceptor	3.49	-1.4	Trp 467, Arg 552, Asp 568, Asp 232, Gly 567, Trp 565, Phe 601, Ile 396, His 626
**C20-3W37**	-8.0	0.94	C	6-ring	Phe 476	H-pi	4.26	-0.6	Ser 474, Trp 432, Asp 232, Lys 506, Asn 475, Met 470
**C21-3W37**	-9.3	1.91	O	O	Tyr 659	H-donor	2.98	-0.9	Met 302, Asp 305, Glu 792, Asn 758, Glu 301, Glu 792, Arg 670, Gly 700, Leu 663
			O	N	Arg 699	H-acceptor	3.18	-1.9	
**C22-3W37**	-9.5	1.93	O	O	Glu 792	H-donor	2.90	-0.9	Arg 670, Arg 699, Leu 663, Glu 301, Tyr 659, Gly 791, Thr 662, Ile 759, Thr 790, Arg 814
			O	N	Glu 792	H-acceptor	3.12	-1.6	
**C23-3W37**	-13.0	1.00	C	O	Asp 357	H-donor	3.21	-1.2	Asp 597, Trp 565, Trp 467, Asp 568, Gly 567, Asp 469, Arg 552, Ile 396, Met 470, His 626, Ile 358
			O	N	Trp 432	H-acceptor	2.99	-2.0	
			C	5-ring	Trp 329	H-pi	3.76	-0.6	
			6-ring	6-ring	Phe 601	Pi-pi	3.99	-0.0	
**D-3W37**	-13.4	1.25	O	O	Asp 568	H-donor	2.83	-0.9	Met 470, Trp 432, Trp 329, Phe 601, Asp 469, Ala 628, Ala 602, Trp 467, His 626, Trp 565, Ile 396, Arg 624, Gly 567, Ile 233, Arg 552, Phe 236, Asn 237
			O	O	Asp 357	H-donor	3.21	-2.1	
			O	O	Asp 232	H-donor	2.98	-0.9	
			C	O	Asp 568	H-donor	3.33	-0.8	

DS: Docking score energy (kcal.mol^{-1}); **RMSD**: Root-mean-square deviation (Å); **L**: Ligand; **P**: Protein; **T**: Type; **D**: Distance (Å); **E**: Energy (kcal.mol^{-1})

Table S4. Detailed molecular docking simulation results for ligands (**C1-C23** and **D**)-**7TAA** inhibitory complexes.

**Ligand-protein complex**			**Hydrogen bond**						**van der Waals interaction**
**Name**	**DS**	**RMSD**	**L**	**P**		**T**	**D**	**E**	
**C1-7TAA**	-8.0	1.25	6-ring	6-ring	Tyr 82	Pi-pi	3.80	-0.0	Trp 83, His 80, His 296, Asp 297, Arg 344, Asp 206, Glu 230, Arg 204
**C2-7TAA**	-8.2	0.99	6-ring	C	Trp 83	Pi-H	3.68	-0.6	His 80, Asp 340, Gln 35, Tyr 75, Gly 167, Val 171
**C3-7TAA**	-12.0	0.70	C<br>O<br>O<br>O	O<br>N<br>N<br>5-ring	Asp 340<br>Arg 344<br>Arg 344<br>His 80	H-donor<br>H-acceptor<br>H-acceptor<br>H-pi	3.50<br>3.39<br>2.92<br>3.98	-0.6<br>-0.7<br>-2.5<br>-0.9	His 296, Tyr 82, Trp 83, Tyr 75, Glm 35
**C4-7TAA**	-7.9	1.03	C	6-ring	Tyr 82	H-pi	4.15	-0.6	His 122, Leu 173, Asp 340, Arg 344, Trp 83, His 80, Asp 206, Leu 166
**C5-7TAA**	-11.0	1.22	O<br>O<br>C	N<br>N<br>5-ring	Arg 344<br>Arg 344<br>His 80	H-acceptor<br>H-acceptor<br>H-pi	3.14<br>3.16<br>3.88	-3.2<br>-2.2<br>-1.1	Trp 83, His 296, Asp 297, Asp 206, Gln 35, Asp 340, Tyr 82
**C6-7TAA**	-9.2	1.03	O<br>O	O<br>N	Asp 340<br>Gln 35	H-donor<br>H-donor	2.71<br>3.09	-4.3<br>-1.7	Tyr 79, Arg 344, Asp 77, Pro 341, Tyr 75, Trp 83, His 80
**C7-7TAA**	-9.3	1.58	O<br>O	O<br>N	Asp 340<br>Gln 35	H-donor<br>H-acceptor	2.93<br>3.14	-3.1<br>-1.2	Tyr 79, Tyr 82, His 80, Arg 344, Trp 83, Tyr 75, Pro 341
**C8-7TAA**	-9.0	2.44	O<br>O	N<br>6-ring	Arg 344<br>Tyr 82	H-acceptor<br>H-pi	3.00<br>4.27	-2.2<br>-0.7	Arg 204, Trp 83, His 80, Asp 206, His 296, Asp 297, Asp 340
**C9-7TAA**	-12.1	0.86	O<br>O<br>O<br>O	O<br>O<br>N<br>O	Asp 340<br>Asp 340<br>Gln 35<br>Tyr 79	H-donor<br>H-donor<br>H-acceptor<br>H-acceptor	2.87<br>2.98<br>3.14<br>2.89	-1.0<br>-0.8<br>-0.8<br>-0.9	Tyr 75, Trp 83, Pro 341, Arg 344, His 80, Asp 77
**C10-7TAA**	-7.9	1.33	O	O	Asp 340	H-donor	3.03	-2.2	His 80, Trp 83, His 296, Tyr 82, Asp 297, Arg 344, Asp 206
**C11-7TAA**	-9.1	1.05	S<br>S	O<br>N	asp 340<br>Gln 35	H-donor<br>H-acceptor	3.77<br>3.95	-1.2<br>-0.7	Tyr 75, Tyr 82, Arg 344, His 80, Trp 83, Tyr 79
**C12-7TAA**	-7.8	1.66	O	O	Asp 206	H-donor	3.03	-2.4	Thr 207, His 122, Tyr 82, Leu 173, His 80, Trp 83, Asp 340, Arg 344, Asp 297, Glu 230
**C13-7TAA**	-11.0	0.92	O<br>O<br>C	N<br>N<br>5-ring	Arg 344<br>Arg 344<br>His 80	H-acceptor<br>H-acceptor<br>H-pi	3.28<br>3.21<br>3.98	-2.7<br>-2.7<br>-0.7	His 296, Trp 83, Asp 297, Asp 206, Asp 340, Tyr 75, Gln 35, Tyr 79, Tyr 82
**C14-7TAA**	-11.3	1.94	O<br>O<br>C	N<br>N<br>5-ring	Arg 344<br>Arg 344<br>His 80	H-acceptor<br>H-acceptor<br>H-pi	3.41<br>3.20<br>4.22	-0.9<br>-1.8<br>-1.2	Asp 297, His 296, Tyr 75, Gln 35, Tyr 82, Asp 340, Arg 204, Trp 83, Asp 206, Glu 230
**C15-7TAA**	-8.9	1.82	O<br>C	N<br>6-ring	His 122<br>Tyr 82	H-acceptor<br>H-pi	3.15<br>3.85	-1.6<br>-0.6	His 80, Trp 83, Asp 297, Arg 344, Gln 35, Asp 340, His 296, Leu 173, Thr 207, Asp 206
**C16-7TAA**	-11.2	0.88	O<br>O<br>O	O<br>N<br>O	Tyr 75<br>Gln 35<br>Tyr 79	H-donor<br>H-acceptor<br>H-acceptor	3.15<br>3.03<br>2.89	-2.5<br>-1.0<br>-1.2	Arg 344, Pro 341, His 80, Asp 340, Trp 83
**C17-7TAA**	-8.8	2.41	O<br>C	N<br>5-ring	Trp 83<br>His 80	H-acceptor<br>H-pi	3.12<br>3.89	-0.8<br>-1.0	Gln 35, Asp 340, Arg 344, Tyr 79, Tyr 82
**C18-7TAA**	-7.8	1.16	C	6-ring	Tyr 75	H-pi	4.64	-0.6	Gln 35, Trp 83, Arg 344, Tyr 82, Asp 340, Leu 173, His 122, Asp 206, His 80
**C19-7TAA**	-9.0	1.95	S<br>S	O<br>N	Asp 297<br>Arg 344	H-donor<br>H-acceptor	3.67<br>3.44	-2.0<br>-4.5	Gln 35, Tyr 75, Asp 340, Trp 83, His 80, Leu 173, Tyr 82
**C20-7TAA**	-7.7	1.29	C	5-ring	His 80	H-pi	4.01	-0.9	Gly 167, Asp 340, Arg 344, Tyr 82, Asp 206, Trp 83, Thr 207, His 122, Leu 166, Leu 173
**C21-7TAA**	-11.3	1.96	O<br>C<br>O	O<br>O<br>O	Tyr 75<br>Asp 340<br>Tyr 79	H-donor<br>H-donor<br>H-acceptor	2.91<br>3.45<br>3.00	-1.0<br>-0.6<br>-0.8	Trp 83, Leu 232, His 210, Glu 230, Tyr 155, Lys 209, Thr 207, Leu 166, Leu 173, Arg 344, Gln 35, His 80
**C22-7TAA**	-11.0	1.69	O<br>O<br>C	N<br>O<br>6-ring	Gln 35<br>Tyr 79<br>Tyr 82	H-acceptor<br>H-acceptor<br>H-pi	3.11<br>3.06<br>3.88	-0.7<br>-0.7<br>-0.8	Typ 83, Asp 206, His 80, Asp 77, Tyr 75, Arg 344, Lys 209, Asp 340, Pro 341, Leu 232, Tyr 155, Leu 166, Glu 230, His 210, Asp 297, Thr 207
**C23-7TAA**	-9.5	1.05	O<br>O	O<br>N	Asp 340<br>Trp 83	H-donor<br>H-acceptor	2.91<br>3.05	-3.4<br>-1.2	Tyr 75, Leu 173, Asp 168, Gly 167, Val 171, Tyr 79, Gln 35, Arg 344, His 80

**D-7TAA**	-12.5	1.88	O	O	Asp 340	H-donor	2.86	-2.2	His 296, Pro 341, Val 171, Trp 83, Asp 297, Arg 344, Leu 173, Tyr 155, Leu 166, Leu 232, His 210, Thr 207, Asp 206, Tyr 82, Tyr 75, Gln 35
			O	O	Glu 230	H-donor	2.91	-3.5	
			O	O	Asp 340	H-donor	3.03	-1.4	
			O	5-ring	His 80	H-pi	4.92	-0.6	

DS: Docking score energy (kcal.mol^{-1}); **RMSD**: Root-mean-square deviation (Å); **L**: Ligand; **P**: Protein; **T**: Type; **D**: Distance (Å); **E**: Energy (kcal.mol^{-1})

Table S5. Physicochemical properties of studied ligands (**C1-C23** and **D**).

Ligands (Compound)	$DS_{average}$ (kcal.mol^{-1})	Mass (amu)	Volume (Å)	Polarisability (Å^{3})	Dispersion cefficients		Total interaction (3W37/7TAA)
					LogP	LogS	Hydrogen bond
**C1**	-8.1	106.0	203.2	14.8	2.91	-2.45	1/ 1
**C2**	-7.6	106.1	202.9	14.9	2.73	-2.41	0/ 1
**C3**	-10.0	130.2	246.3	14.6	2.46	-1.58	1/ 4
**C4**	-8.1	136.3	270.8	19.2	2.35	-2.37	1/ 1
**C5**	-10.0	144.2	273.4	17.5	2.52	-1.82	2/ 3
**C6**	-9.2	88.0	150.0	9.2	-0.47	0.56	2/ 2
**C7**	-8.6	86.2	171.6	10.7	1.79	-0.59	1/ 2
**C8**	-10.1	140.4	267.4	18.6	1.84	-1.60	3/ 2
**C9**	-10.5	116.3	204.6	14.2	0.39	-0.19	2/ 2
**C10**	-8.3	100.2	199.5	13.6	1.88	-0.98	2/ 4
**C11**	-11.5	126.4	178.9	14.9	1.93	-2.31	7/ 1
**C12**	-10.1	146.2	252.2	15.1	0.91	-0.72	4/ 2
**C13**	-11.6	172.3	334.3	20.8	3.89	-2.80	4/ 2
**C14**	-9.6	186.4	359.4	20.9	4.01	-3.42	1/ 3
**C15**	-9.1	170.3	317.3	21.3	3.67	-2.58	2/ 2
**C16**	-10.2	46.3	68.2	4.3	-0.28	0.50	2/ 1
**C17**	-8.6	110.8	175.7	12.6	0.75	-0.74	1/ 2
**C18**	-8.0	162.4	320.9	20.9	4.92	-4.08	1/ 1
**C19**	-10.4	176.3	301.4	20.8	2.68	-1.93	3/ 2
**C20**	-7.9	204.5	383.4	28.2	4.91	-4.29	1/ 1
**C21**	-10.3	256.6	485.6	31.2	4.96	-4.28	2/ 3
**C22**	-10.3	254.5	312.0	24.8	5.02	-4.17	2/ 3
**C23**	-11.3	152.3	212.9	17.7	1.78	-1.39	4/ 2
**D**	-13.0	267.8	297.2	25.3	2.69	-1.52	5/ 4

Table S6. Pharmacokinetic and pharmacological properties of compounds **C1-12**.

Property	C1	C2	C3	C4	C5	C6	C7	C8	C9	C10	C11	C12	Unit
**Absorption**													
Water solubility	-2.522	-2.522	-1.296	-3.568	-1.909	0.575	-0.5	-2.122	0.14	-1.082	-1.837	-0.04	(1)
Caco2 permeability	1.547	1.547	1.628	1.401	1.606	1.578	1.482	1.508	1.49	1.48	1.393	1.211	(2)
Intestinal absorption (human)	95.713	95.713	96.332	95.898	95.372	95.666	93.284	96.981	94.195	93.765	93.461	86.33	(3)
Skin Permeability	-1.236	-1.236	-2.25	-1.721	-2.025	-3.586	-2.429	-2.34	-3.323	-2.109	-1.979	-3.193	(4)
P-glycoprotein substrate	No	No	No	Yes	No	No	Yes	Yes	No	No	Yes	No	(5)
P-glycoprotein I inhibitor	No	No	No	No	No	No	No	No	No	No	No	No	(5)
P-glycoprotein II inhibitor	No	No	No	No	No	No	No	No	No	No	No	No	(5)
**Distribution**													
VDss (human)	0.325	0.325	-0.095	0.396	-0.004	-0.188	-0.036	0.153	-0.177	0.024	0.02	-0.243	(6)
Fraction unbound (human)	0.362	0.362	0.596	0.48	0.543	0.78	0.67	0.565	0.742	0.615	0.658	0.704	(6)
BBB permeability	0.409	0.409	0.402	0.732	0.454	-0.259	0.092	0.267	-0.215	0.16	0.262	-0.204	(7)
CNS permeability	-1.677	-1.677	-2.191	-2.37	-2.343	-2.835	-2.324	-2.535	-2.81	-2.25	-2.588	-2.639	(8)
**Metabolism**													
CYP2D6 substrate	No	No	No	No	No	No	No	No	No	No	No	No	(5)
CYP3A4 substrate	No	No	No	No	No	No	No	No	No	No	No	No	(5)
CYP1A2 inhibitior	No	No	No	No	No	No	No	No	No	No	No	No	(5)
CYP2C19 inhibitior	No	No	No	No	No	No	No	No	No	No	No	No	(5)
CYP2C9 inhibitior	No	No	No	No	No	No	No	No	No	No	No	No	(5)
CYP2D6 inhibitior	No	No	No	No	No	No	No	No	No	No	No	No	(5)
CYP3A4 inhibitior	No	No	No	No	No	No	No	No	No	No	No	No	(5)
**Excretion**													
Total Clearance	0.254	0.264	0.455	0.213	0.558	0.676	0.356	0.217	0.741	0.452	0.233	0.808	(9)
Renal OCT2 substrate	No	No	No	No	No	No	No	No	No	No	No	No	(5)
**Toxicity**													
AMES toxicity	No	No	No	No	No	No	No	No	No	No	No	No	(5)
Max. tolerated dose (human)	0.921	0.921	0.938	0.777	0.74	1.268	1.12	0.848	1.169	0.997	0.891	1.152	(10)
hERG I inhibitor	No	No	No	No	No	No	No	No	No	No	No	No	(5)
hERG II inhibitor	No	No	No	No	No	No	No	No	No	No	No	No	(5)
Oral Rat Acute Toxicity (LD50)	1.841	1.841	1.766	1.88	1.884	1.903	1.799	1.891	1.837	1.735	2.971	1.775	(11)
Oral Rat Chronic Toxicity (LOAEL)	2.168	2.168	2.411	2.336	2.323	2.504	1.698	2.017	2.446	1.767	1.623	2.522	(12)
Hepatotoxicity	No	No	No	No	No	No	No	No	No	No	No	No	(5)
Skin Sensitisation	No	No	Yes	Yes	Yes	No	No	Yes	Yes	Yes	No	No	(5)
T.Pyriformis toxicity	-0.022	-0.022	-0.188	0.579	0.027	-1.545	-1.124	0.087	-0.963	-0.815	0.795	-0.288	(13)
Minnow toxicity	1.31	1.31	1.361	1.203	1.166	2.717	2.177	1.653	2.595	1.902	1.593	2.499	(14)

(1) log mol.L^{-1}; (2) log Papp (10^{-6} cm.s^{-1}); (3) %; (4) log Kp; (5) Yes/No; (6) log L.kg^{-1}; (7) log BB; (8) log PS;
(9) log mL.min^{-1}.kg^{-1}; (10) log mg.kg^{-1}.day^{-1}; (11) mol.kg^{-1}; (12) log mg.kg^{-1}_bw.day^{-1}; (13) log µg.L^{-1}; (14) log mM

Table S7. Pharmacokinetic and pharmacological properties of compounds **C13-23** and **D**.

Property	C13	C14	C15	C16	C17	C18	C19	C20	C21	C22	C23	D	Unit
**Absorption**													
Water solubility	-3.136	-3.417	-2.51	0.83	-0.738	-5.017	-2.002	-6.033	-5.562	-5.477	-1.881	-1.482	(1)
Caco2 permeability	1.604	1.575	1.233	1.554	1.61	1.541	1.612	1.423	1.558	1.565	1.202	-0.481	(2)
Intestinal absorption (human)	94.665	94.145	94.961	95.367	97.242	94.759	92.538	96.219	92.004	92.51	89.457	4.172	(3)
Skin Permeability	-1.694	-1.625	-1.739	-2.93	-2.569	-1.204	-2.704	-1.464	-2.717	-2.715	-2.716	-2.735	(4)
P-glycoprotein substrate	No	No	No	No	Yes	No	No	No	No	No	No	Yes	(5)
P-glycoprotein I inhibitor	No	No	No	No	No	No	No	No	No	No	No	No	(5)
P-glycoprotein II inhibitor	No	No	No	No	No	No	No	No	No	No	No	No	(5)
**Distribution**													
VDss (human)	0.115	0.106	0.019	-0.641	-0.082	0.787	0.005	0.704	-0.543	-0.574	-0.009	-0.836	(6)
Fraction unbound (human)	0.424	0.359	0.47	0.814	0.61	0.13	0.567	0.158	0.101	0.104	0.49	0.505	(6)
BBB permeability	0.608	0.621	0.601	-0.337	-0.023	0.653	0.466	0.831	-0.111	-0.084	-0.222	-1.717	(7)
CNS permeability	-2.214	-1.953	-2.179	-2.644	-2.801	-1.372	-2.558	-1.808	-1.816	-1.763	-2.185	-6.438	(8)
**Metabolism**													
CYP2D6 substrate	No	No	No	No	No	No	No	No	No	No	No	No	(5)
CYP3A4 substrate	No	No	No	No	No	No	No	No	Yes	Yes	No	No	(5)
CYP1A2 inhibitior	No	No	No	No	No	Yes	No	No	No	No	No	No	(5)
CYP2C19 inhibitior	No	No	No	No	No	No	No	No	No	No	No	No	(5)
CYP2C9 inhibitior	No	No	No	No	No	No	No	No	No	No	No	No	(5)
CYP2D6 inhibitior	No	No	No	No	No	No	No	No	No	No	No	No	(5)
CYP3A4 inhibitior	No	No	No	No	No	No	No	No	No	No	No	No	(5)
**Excretion**													
Total Clearance	1.64	1.514	0.602	0.498	0.682	0.323	0.426	0.067	1.763	1.817	0.709	0.428	(9)
Renal OCT2 substrate	No	No	No	No	No	No	No	No	No	No	No	No	(5)
**Toxicity**													
AMES toxicity	No	No	No	No	Yes	No	No	No	No	No	Yes	No	(5)
Max. tolerated dose (human)	0.567	0.696	0.773	1.469	1.081	0.917	0.783	0.177	-0.708	-0.713	0.769	0.435	(10)
hERG I inhibitor	No	No	No	No	No	No	No	No	No	No	No	No	(5)
hERG II inhibitor	No	No	No	No	No	No	No	No	No	No	No	Yes	(5)
Oral Rat Acute Toxicity (LD50)	1.803	1.651	1.7	1.886	2.356	1.753	2.029	1.589	1.44	1.449	1.764	2.449	(11)
Oral Rat Chronic Toxicity (LOAEL)	2.449	2.416	2.266	2.591	1.861	2.508	2.045	1.391	3.181	3.109	2.305	5.319	(12)
Hepatotoxicity	No	No	No	No	No	No	No	No	No	No	No	No	(5)
Skin Sensitisation	Yes	Yes	Yes	No	Yes	Yes	Yes	No	Yes	Yes	No	No	(5)
T.Pyriformis toxicity	0.848	1.349	0.753	-1.075	-0.528	1.715	0.594	1.772	0.84	0.865	0.367	0.285	(13)
Minnow toxicity	0.584	0.623	0.855	2.887	2.015	0.018	1.247	0.107	-1.083	-0.956	1.778	16.823	(14)

(1) log mol.L^{-1}; (2) log Papp (10^{-6} cm.s^{-1}); (3) %; (4) log Kp; (5) Yes/No; (6) log L.kg^{-1}; (7) log BB; (8) log PS;
(9) log mL.min^{-1}.kg^{-1}; (10) log mg.kg^{-1}.day^{-1}; (11) mol.kg^{-1}; (12) log mg.kg^{-1}_bw.day^{-1}; (13) log µg.L^{-1}; (14) log mM

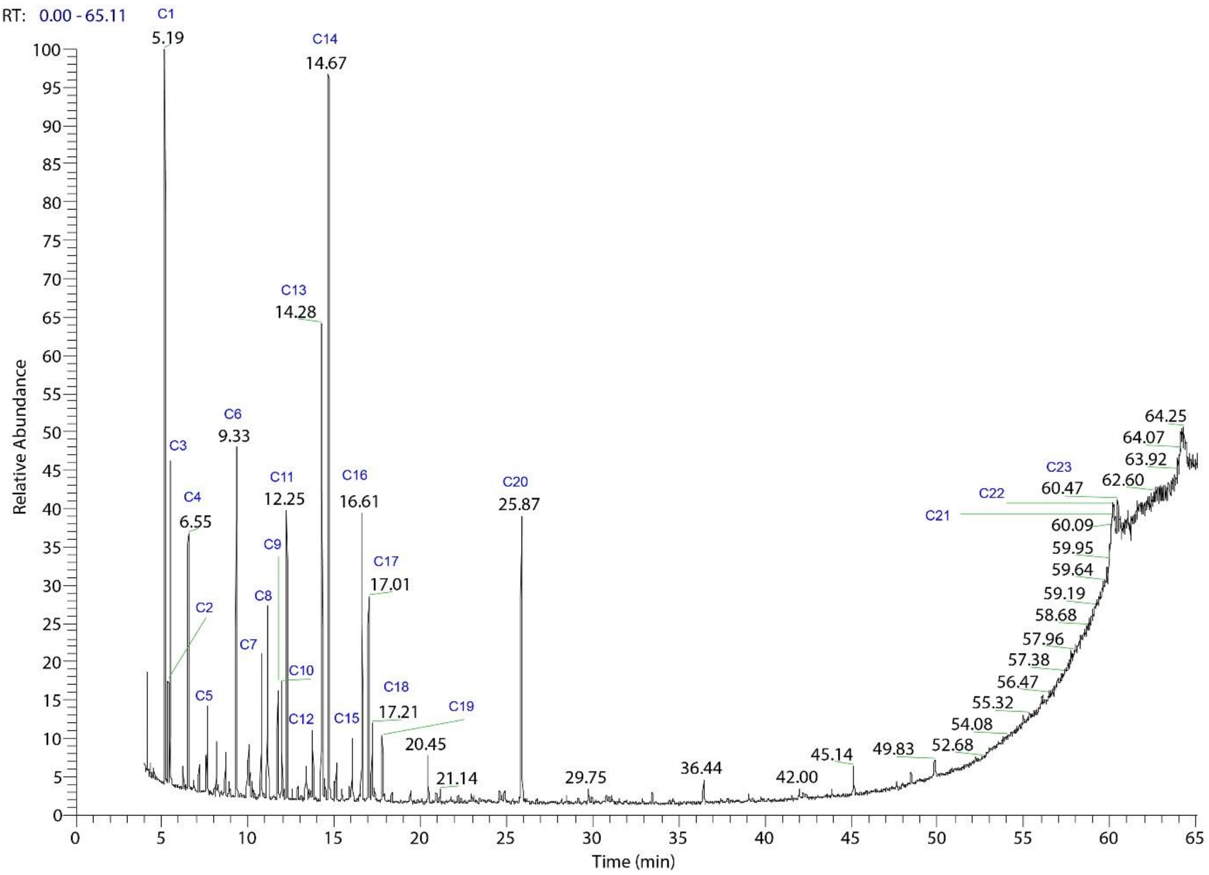

Fig. S1. GC-MS chromatogram of the fungal extract.

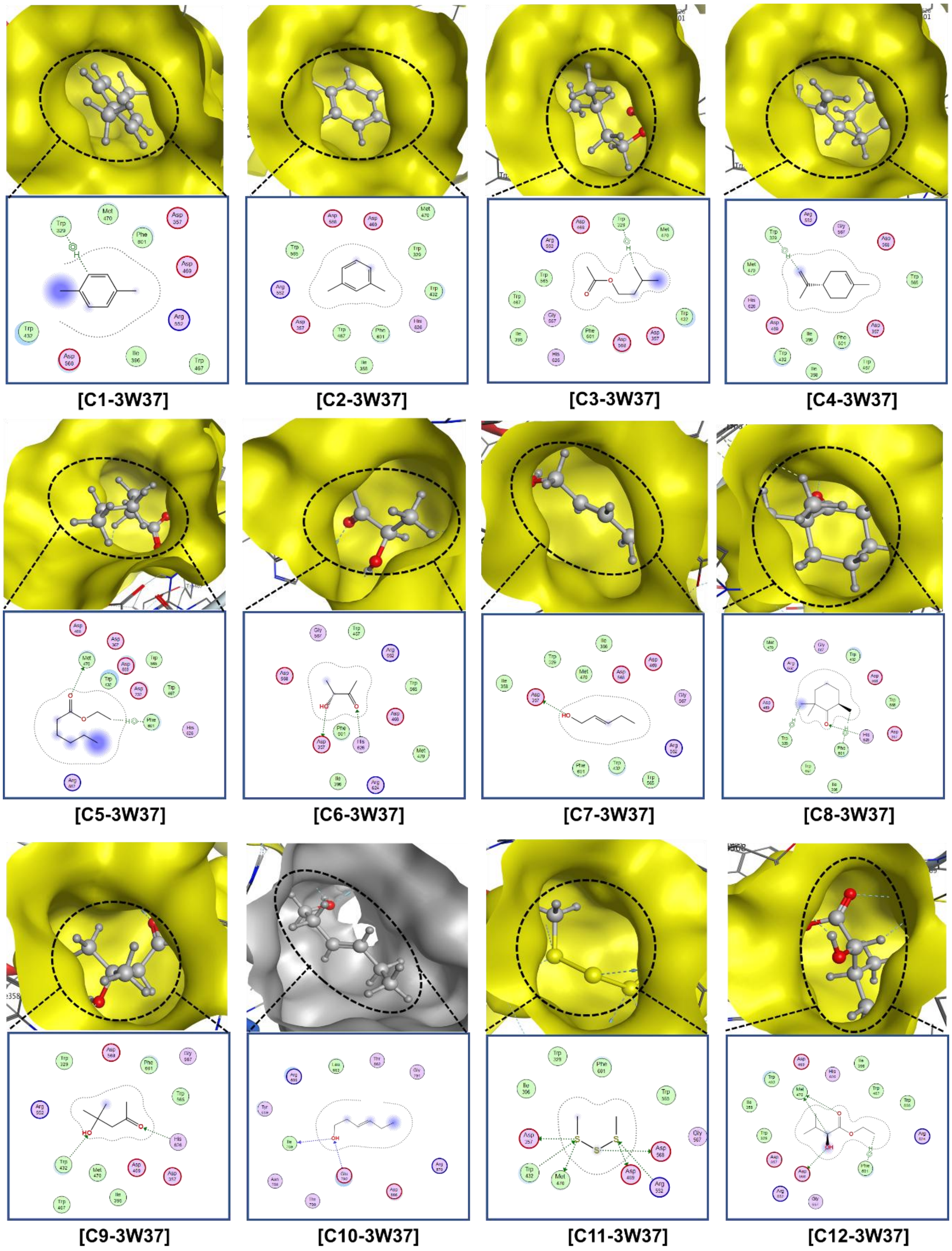

Fig. S2. Visual presentation and in-pose interaction map of ligand-**3W37** (ligand: **C1-C12)** inhibitory structures.

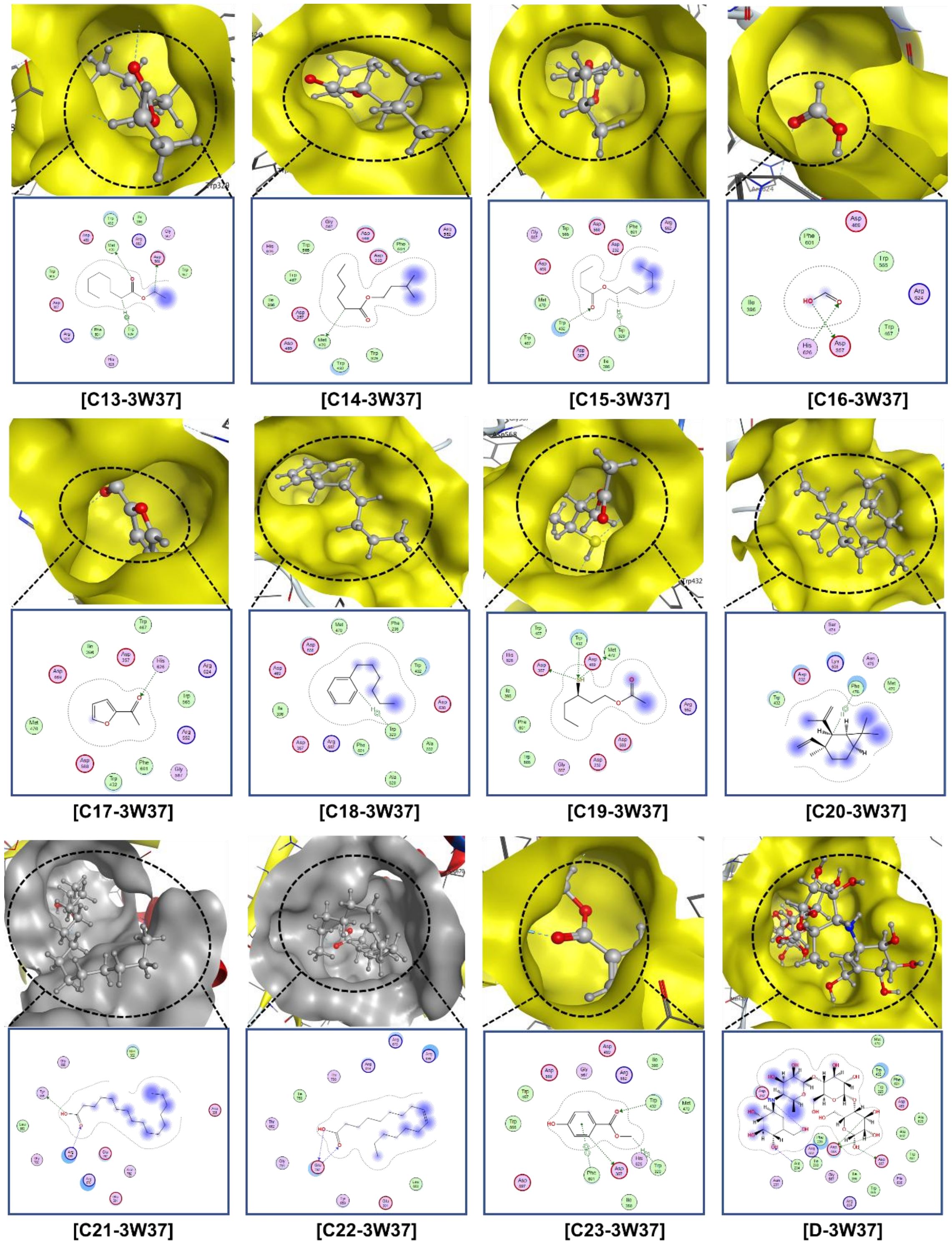

Fig. S3. Visual presentation and in-pose interaction map of ligand-**3W37** (ligand: **C13-C23** and **D)** inhibitory structures.

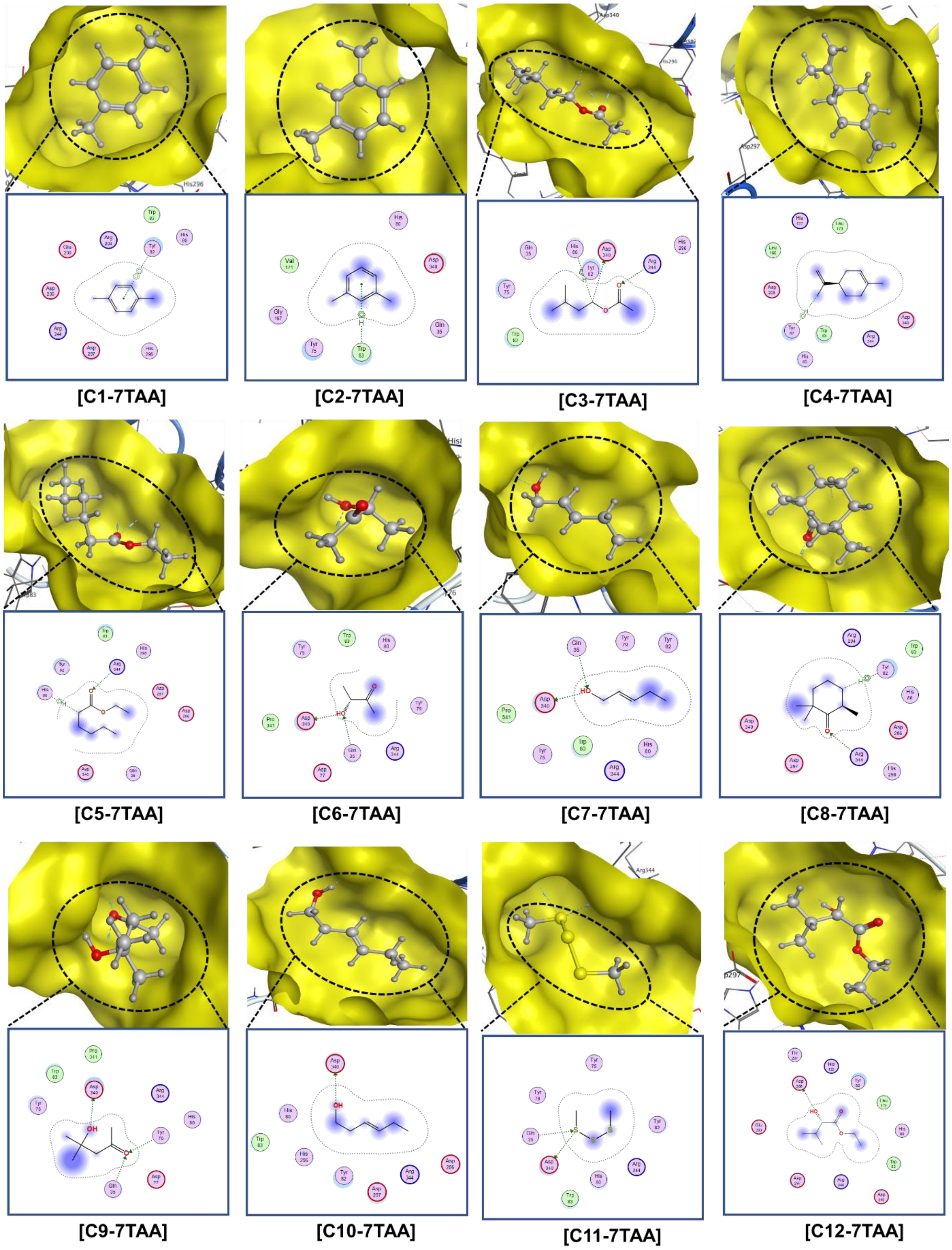

Fig. S4. Visual presentation and in-pose interaction map of ligand-**7TAA** (ligand: **C1-C12)** inhibitory structures.

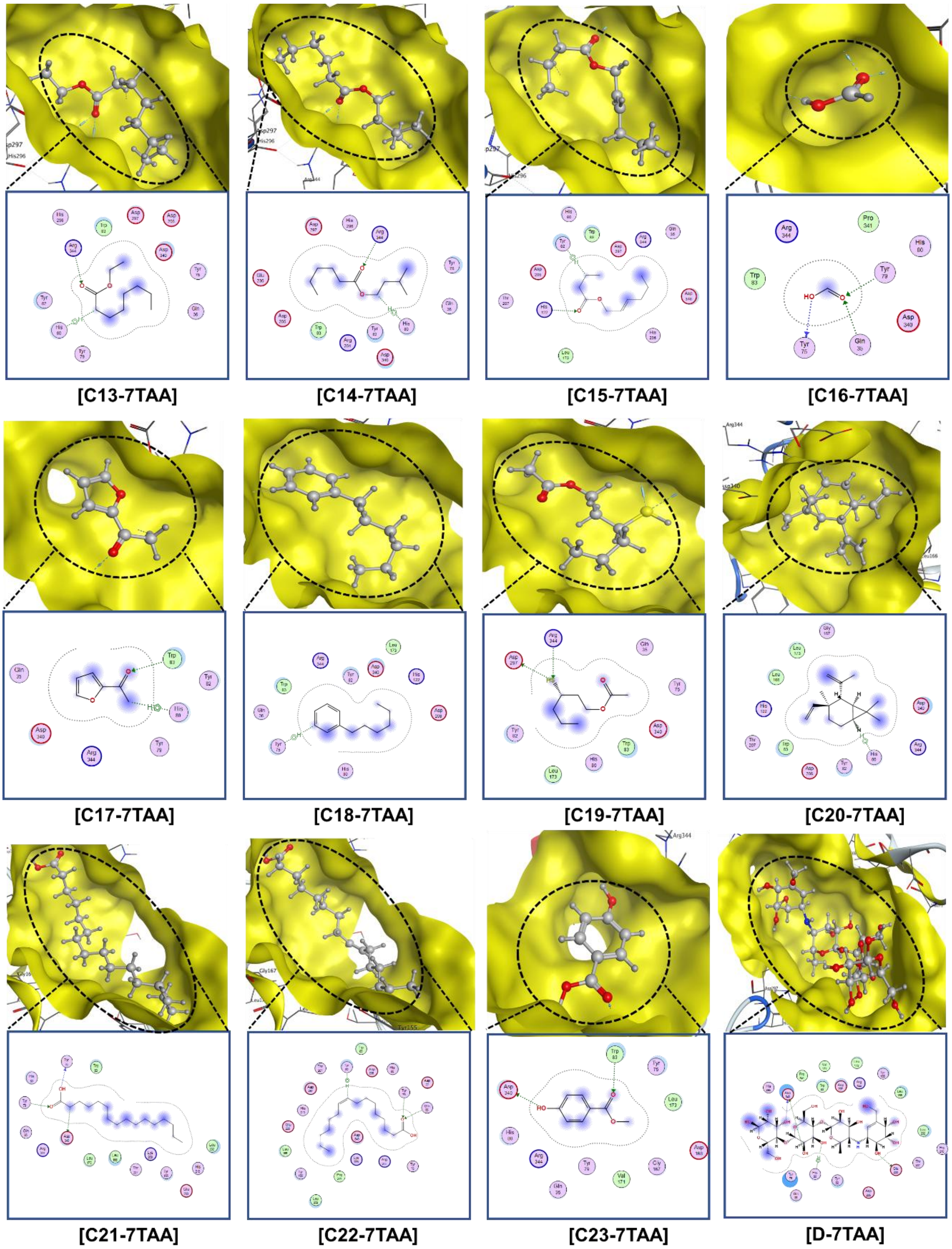

Fig. S5. Visual presentation and in-pose interaction map of ligand-**7TAA** (ligand: **C13-C23** and **D)** inhibitory structures.

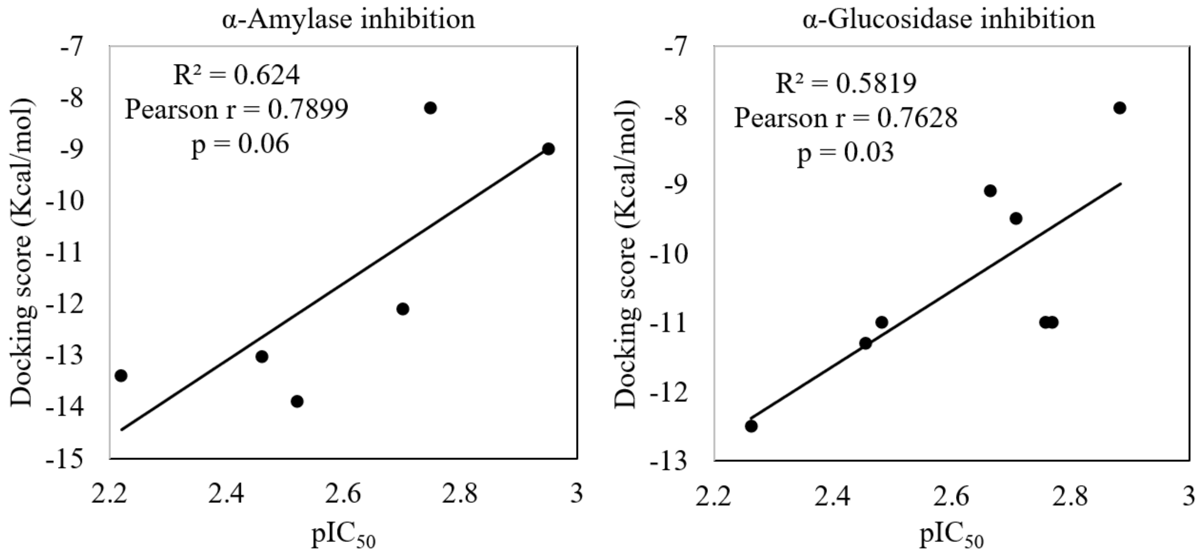

Fig. S6. Correlation between docking score and experimental bioactivity.
